# Estimating Vascular Age to Evaluate the Association Between Aging and Cardiovascular Disease

**DOI:** 10.1111/acel.70503

**Published:** 2026-05-05

**Authors:** Yueqi Lu, Yucong Zhang, Bangwei Chen, Lei Ruan, Yaxin Li, Linpeng Wang, Shida Zhu, Tao Li, Li Luo, Cuntai Zhang, Yutao Du

**Affiliations:** ^1^ BGI Genomics, BGI‐Shenzhen Shenzhen China; ^2^ Department of Geriatrics, Tongji Hospital, Tongji Medical College Huazhong University of Science and Technology Wuhan China; ^3^ College of Life Sciences University of Chinese Academy of Sciences Beijing China; ^4^ BGI, BGI‐Shenzhen Shenzhen China; ^5^ School of Basic Medicine Hebei Medical University Shijiazhuang Hebei China; ^6^ Center of Forensic Expertise Affiliated Hospital of Zunyi Medical University Zunyi Guizhou China

**Keywords:** biological age, cardiovascular disease, retrospective cohort study, vascular aging

## Abstract

Vascular aging, characterized by progressive structural and functional deterioration of the vasculature, serves as a critical pathophysiological nexus between chronological aging and cardiovascular disease (CVD). This study establishes a quantitative vascular age model to decode individualized vascular senescence patterns, thereby enabling early identification of accelerated aging phenotypes for targeted intervention. We collected physical examination records from 2009 to 2019 and a total of 8578 participants aged 20–70 years were enrolled in this study. We constructed sex‐specific basic vascular age models based on healthy individuals by Klemera‐Doubal method and calculated the normalized cardiovascular age acceleration (NCAA, *η*) as an estimate of vascular aging status. The association between *η* and CVD risk were evaluated across subgroups. Furthermore, we developed expanded models by incorporating traditional CVD risk factors that were significantly associated with *η* index. Male with lower values of *η*, which meant relatively higher vascular aging velocity, had a higher risk of CVD adjusted by chronological age (HR = 1.21, 95% CI = 1.01–1.45). In subgroup analysis, *η* index exhibited age‐ and sex‐specific associations with traditional CVD risk factors. After adding body mass index, fasting blood glucose, and triglycerides significantly related to *η* in male, the CVD prediction by expand *η* were improved in age‐adjusted model (HR = 1.25, 95% CI = 1.04–1.50). The vascular age model emerges as a robust composite biomarker for CVD risk stratification. Our findings establish an evidence‐based framework for precision prevention, prioritizing high‐risk phenotypes for early intervention to mitigate CVD burden.

## Graphical abstract

The normalized cardiovascular age acceleration (NCAA) index is estimated using the Klemera‐Doubal method (KDM) based on annual physical examination records at Tongji Hospital in Wuhan, China. Vascular function‐based (basic) NCAA shows different associations with cardiometabolic risk factors by age and sex. Basic and expanded NCAA are associated with a 21%–25% increased risk of CVD development.

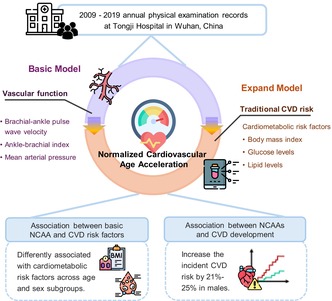

## 1 Introduction

Cardiovascular disease (CVD) is the leading cause of mortality worldwide, contributing significantly to its global burden (Collaborators 2023). Vascular aging (VA) is an independent risk factor of CVD, which increases dramatically with age in most but not all people (Mitchell 2021). Arterial stiffness, measured as pulse wave velocity (PWV), is a key clinical feature of VA. Moreover, arterial stiffness is associated with temporal biological alterations in the arteries, elevating the risk factors for cardiovascular injury (Kucharska‐Newton et al. 2019). Schutte et al. further demonstrated that early VA reflecting increased arterial stiffness at younger chronological ages (CA) was a robust predictor of cardiovascular outcomes (Schutte et al. 2020). Given the tight relationship between the morbidity and mortality of CVD and age, identifying high‐risk populations for VA during the aging process may be an effective strategy.

Aging is a natural biological process in which physical and physiological functions decline, leading to increased susceptibility to CVD (Damluji et al. 2024). As the age increases, excess reactive oxygen species (ROS) accumulation, low‐grade inflammatory status, cellular senescence, and loss of protein homeostasis jointly promote CVD risk in elderly people (Agnoletti et al. 2022; Evans et al. 2020; Ferrucci and Fabbri 2018; Hu et al. 2022; Rizvi et al. 2021; Shen et al. 2018; Toth et al. 2020; Ungvari et al. 2018). Previous study indicated that CA may not accurately distinguish the actual aging status, nor can it identify differences in the true aging state among individuals of the same CA (Elliott et al. 2021). Recently, biological age (BA) has been promoted as a better index of an individual's overall health. Multiple measurements of BA have been developed, such as methylation levels, telomere length, proteome, and clinical biomarkers (Jylhava et al. 2017; Oh et al. 2023). BA model, derived from clinical biomarkers, is known for its superior accessibility, standardization, and practicality (Dai et al. 2025).

The Klemera‐Doubal method (KDM) is a popular method to calculate BA (Klemera and Doubal 2006). KDM stands out over the multiple linear regression (MLR) method and Hochschild's method by treating CA as an independent factor, preventing it from covering the age‐related biomarkers' effect, and using an advanced algorithm (Jia et al. 2017). Heart age from the Framingham study is defined as the CA of a person with the same CVD risk score but other risk factors at the normal level (D'Agostino Sr. et al. 2008). Recently, Alessandro Gialluisi et al. used Deep Neural Networks based on 36 circulating biomarkers to estimate BA for predicting CVD death and hospitalization (Gialluisi et al. 2022). Compared to CA, BA calculated from clinical biomarkers in healthy populations can more precisely reflect an individual's physiological condition and the risk of developing age‐related diseases and mortality (Chen et al. 2024). However, the relationship between VA and CVD development through BA has yet to be extensively explored.

In this study, we estimated basic vascular age from KDM models based on vascular structure and function parameters, including brachial‐ankle pulse wave velocity (baPWV), to investigate the relationship of VA with CVD. Moreover, we developed expanded vascular age models grasping additional information from metabolic factors to improve CVD prediction performance.

## 2 Methods

### 2.1 Study Population and Data Sources

**FIGURE 1:**
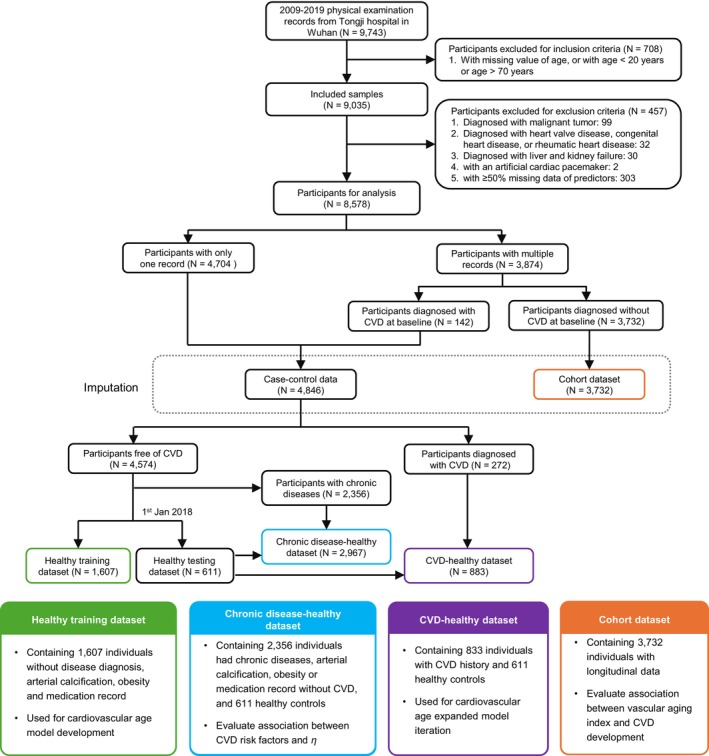
Flow chart of data processing.

This study used data from annual physical examinations at Tongji Hospital in Wuhan China (2009–2019). We enrolled participants with age 20–70 years and excluded those who had malignant tumors, heart valve disease, congenital heart disease, rheumatic heart disease, liver and kidney failure. Moreover, we used CVD as the outcome, including coronary heart disease (CHD) and stroke. CHD was defined by the diagnosis of angina, echocardiography or angiography results, self‐report history of CHD, and related surgical treatment (coronary artery bypass grafting, coronary stents, and percutaneous transluminal coronary angioplasty). The definition of stroke depended on the self‐report history of cerebral infarction or cerebral hemorrhage. Finally, the participants were divided into 4 datasets: healthy training dataset, chronic disease‐healthy dataset, CVD‐healthy dataset, and cohort dataset (Figure 1). The healthy training dataset was for participants with only one record and free of chronic diseases. The chronic disease‐healthy dataset included health controls and non‐CVD cases diagnosed with chronic obstructive pulmonary disease, hypertension, hyperlipidemia, diabetes, thyroid disease, lower extremity arterial blockage (ankle‐brachial index or ABI < 0.9), arterial calcification (ABI ≥ 1.4), obesity (BMI ≥ 28 kg/m^2^), and receiving related medications. CVD‐healthy dataset contained health controls and CVD individuals. The cohort dataset comprised participants with multiple follow‐up records and no diagnosis of CVD at baseline. This study was approved by the Tongji Hospital Medical Ethics Committee (TJ‐IRB20191215). All participants signed the informed consent.

### 2.2 Demographic Information and Clinical Parameters Collection

Participants underwent face‐to‐face interviewer‐administered to collect demographic information (age, sex, BMI, disease, and medical history). Non‐invasive examinations, including mean arterial pressure (MAP), heartbeat, baPWV, and ABI were obtained. MAP was calculated using systolic blood pressure (SBP) and diastolic blood pressure (DBP) with the formula $\mathrm{MAP}=\mathrm{DBP}+\left(\mathrm{SBP}-\mathrm{DBP}\right)/3$. Furthermore, total cholesterol (TC), high‐density lipoprotein cholesterol (HDL‐C), low‐density lipoprotein cholesterol (LDL‐C), triglycerides (TG), fasting blood glucose (FBG), glycated hemoglobin (HbA1c), neutrophil‐to‐lymphocyte ratio (NLR) and erythrocyte sedimentation rate (ESR) were measured. Individuals with incomplete data (lacking age or missing > 50% of above parameters) were excluded.

### 2.3 Basic Vascular Age Estimation Model Development

We developed vascular aging estimates using the KDM algorithm in the healthy training dataset to capture normal aging patterns. According to the procedure proposed by a previous study, we constructed models by sex (Li et al. 2020). The first (basic) estimate was based solely on vascular functional indices, baPWV, ABI, and MAP, to provide an integrated assessment of vascular aging from a functional perspective. Specifically, baPWV indicates vascular stiffness, ABI reflects arterial obstruction, and MAP affects cardiac output and systemic vascular resistance. These three non‐invasive vascular measurements collectively demonstrate the central and peripheral circulation and the overall cardiovascular health related to organ perfusion (Aminuddin et al. 2025; DeMers and Wachs 2026). Firstly, these indicators were standardized using the z‐score method and performed PCA to obtain three new independent biomarkers. Then, the basic vascular age was estimated on principal components by using the KDM method (Equation 1). KDM estimates the relationship between CA and the indicator separately, producing the parameters slope *k*, intercept *q*, and residual standard error *s* from the regression model. These relationships are then integrated using a weighted algorithm to derive an optimal estimate of BA (Klemera and Doubal 2006). R package *bioage* was used for model development (Bartlett 2018). To demonstrate the aging status accounting for differences in scale across ages, the normalized cardiovascular age acceleration (NCAA, *η*) was calculated as the residual (CA‐BA) divided by CA (Equation 2) (Rahman and Adjeroh 2019). A lower value of *η* indicated that an individual's cardiovascular age is older than expected based on their CA, suggesting a quicker vascular aging tendency.

(1) $$\text{Cardiovascular}\;\mathrm{age}=\frac{\sum_{j=1}^{m}\left(t_{j}-q_{j}\right)\frac{k_{j}}{{s_{j}}^{2}}}{\sum_{j=1}^{m}{\left(\frac{k_{j}}{s_{j}}\right)}^{2}}$$

(2) $$\eta =\frac{\text{Chronological}\;\mathrm{age}-\text{Cardiovascular}\;\mathrm{age}}{\text{Chronological}\;\mathrm{age}}$$

*t*, The value of a PC from the sample; *k*, *q*, *s*, The parameters obtained from KDM models. *m*, The number of PCs included in the model; *j*: The *j* ^th^ PC included in the model.

In the cohort dataset, we estimated the basic vascular age at baseline, calculated *η*_basic_, and evaluated its association with CVD development. The hazard ratios (HRs) for *η*_basic_ were assessed using Cox proportional hazards models, both as a continuous variable and by tertile categories. The log‐rank test was used to compare the difference in CVD occurrence between different *η*_basic_ groups. Moreover, we evaluated the performance of vascular age models by different age groups. Using the restricted cubic spline (RCS) method, we determined the age cutoff from the association between CA and CVD risk. Additionally, the associations between *η*_basic_ and other incident chronic diseases, including hypertension, type II diabetes, hyperlipidemia, and kidney dysfunction (estimated glomerular filtration rate, eGFR < 60 mL/min/1.73m^2^), were investigated.

### 2.4 Expanded Vascular Model

We then developed the second (expand) estimate upon the basic model by further incorporating traditional CVD risk factors. This expand estimate aimed to provide a more comprehensive representation of vascular aging and improve CVD risk prediction. CVD‐related biomarkers contained metabolic indicators (BMI, TC, HDL‐C, LDL‐C, TG, FBG, and HbA1c), inflammatory indicators (ESR and NLR), and heartbeat. The associations of *η*_basic_ groups and these CVD‐related biomarkers in different age groups were observed. Before calculating aging measures, non‐normally distributed biomarkers (TG, FBG, HbA1c, ESR, and NLR) were log‐transformed. The intersection of *η*_basic_‐related traditional CVD risk factors (*p*‐value < 0.05) across age groups was selected for the expanded vascular model as additional candidate biomarkers. We developed a series of nested models in the training dataset by sequentially adding one additional biomarker at a time. We then evaluated the association between *η*_nested_ and CVD using age‐adjusted logistic regression in the CVD‐healthy dataset, with 100 bootstrap replicates. The optimal model was defined as the vascular model that maximized the OR per SD increase in *η*_nested_ values for CVD compared to the basic model until no further improvement was observed. We calculated the final expanded vascular estimate, *η*_expand_, based on the optimal model and assessed its performance in predicting CVD development in the cohort dataset.

### 2.5 Statistical Analysis

Descriptive and inferential analyses were conducted by using R version 4.3.1. The correlation between estimated cardiovascular age and CA was assessed using Pearson's correlation coefficient. A linear regression model without an intercept was fitted, in which the estimated slope was used to quantify proportional agreement between cardiovascular age and CA. Nonlinear relationships were estimated in 5‐knot restricted cubic spline function models using the *rms* package (Harrell 2023). Difference tests for traditional CVD risk factors across *η* tertile groups in each age subgroup were evaluated by a one‐way ANOVA test for normally distributed continuous variables or a Kruskal‐Wallis rank‐sum test for non‐normally distributed continuous variables through the *tableone* package (Bartel 2022). Jonckheere‐Terpstra tests were used to evaluate the monotonic trends of these risk factors across *η* tertile groups via the *PMCMRplus* package (Pohlert 2024). We performed data imputation on a single‐record dataset and a multi‐record dataset in each sex by random forest method. Vascular functional indices, traditional CVD risk factors for vascular aging estimation, and histories of non‐CVD chronic diseases and related medication were included for imputation. Imputation was conducted with the R package *mice* with 30 iterations (van Buuren and Groothuis‐Oudshoorn 2011). Candidate predictors with skewed distributions were adjusted by log transformation. Then, we also standardized the predictors with means and standard deviations. The parameters of standardization were calculated in the training dataset and used for other datasets.

## 3 Results

We totally collected physical examination records of 9743 participants from Tongji Hospital in Wuhan. After inclusion and exclusion, participants were divided into four datasets (Figure 1), healthy training dataset (*N* = 1607), chronic disease‐healthy dataset (*N* = 2967), CVD‐healthy dataset (*N* = 883), and cohort dataset (*N* = 3732) with mean ages of 49.6 years in males and 46.5 years in females. Males had a higher proportion than females in each dataset. Characteristics of these datasets were shown in Tables S1 and S2, and those of the imputed case–control and cohort datasets are presented in Tables S3–S5. TG, FBG, HbA1c, NLR, and ESR with non‐normal distribution were adjusted by log transformation. Parameters used for *Z*‐score standardization on all predictors were provided in Table S6 and the distributions of these predictors across age groups by sex were demonstrated in Figure S1.

### 3.1 Faster Vascular Aging Was Associated With a Higher Risk of CVD

**FIGURE 2:**
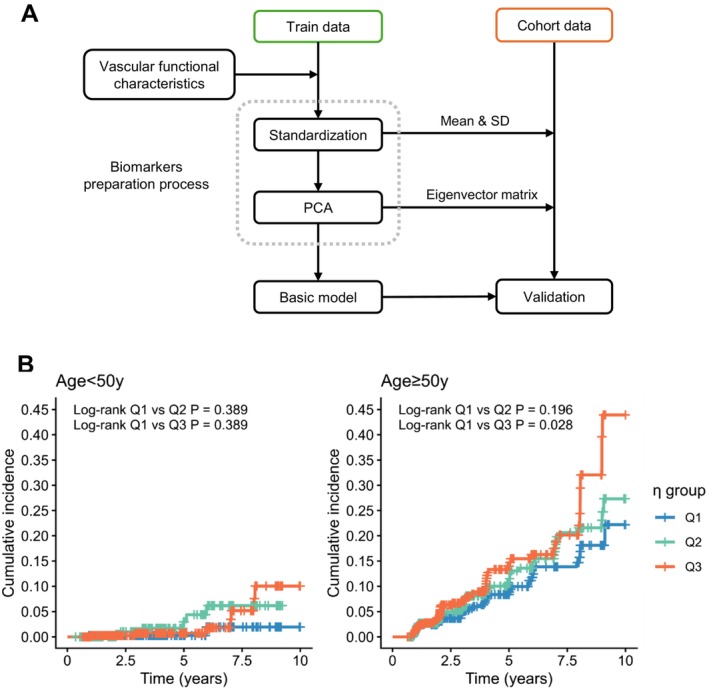
Cardiovascular age basic model development and performance in males. (A) Scheme for basic model development and validation. (B) Association between *η*_basic_ and 10‐year CVD incidence in age < 50 years and age ≥ 50 subgroups of males. CVD incidences were observed from three tertiles of *η*_basic_ values: Q1 ranging from [0.112, 2.450], Q2 from [−0.557, 0.112), and Q3 from [−5.520, −0.557). Log‐rank tests were used to compare the differences in CVD incidence between tertile groups.

We developed the basic vascular models with baPWV, ABI, and MAP in a healthy training dataset by each gender (Figure 2A). Parameters for calculating *η*_basic_ with these three indicators by using KDM methods were listed in Tables S7 and S8. Significant correlations between CA and cardiovascular age estimated from the basic model were shown in the healthy control dataset (Figure S2A). We observed a negative relationship between *η*_basic_ and CA in females (*p*‐value < 0.001), but not in males at cohort baseline (Pearson test *p*‐value = 0.985) (Figure S3). In Cox regression models, lower *η*_basic_ values modestly increased the risk of CVD in males with HR = 1.21 (*p*‐value = 0.038) adjusted by age. Compared to the tertile 1 (Q1) group, participants in the tertile 3 (Q3) group (HR = 1.71, *p*‐value = 0.006) with smaller *η*_basic_ values had higher CVD risk (Table 1). However, *η*_basic_ values were nominally associated with the risk of CVD without age adjustment, and the association was no longer significant after adjusting for age in females. Additionally, no non‐linear relationships between *η*_basic_ values and CVD risk were observed in both males (nonlinear *p*‐value = 0.511) and females (nonlinear *p*‐value = 0.741) in the models adjusted by CA.

**TABLE 1:** Association between normalized cardiovascular age acceleration index **η**_**basic**_ and incident CVD by sex.

Case/N	*η*_basic_				
		Crude		Adjusted^a^	
		HR (95% CI)	*p*	HR (95% CI)	*p*
*Male*					
174/2819	Continuous, per 1 decrease	1.09 (0.92, 1.30)	0.309	1.21 (1.01, 1.45)	0.038
	Multicategory				
46/930	Q1 [0.112, 2.450]	Reference	Reference	Reference	Reference
67/959	Q2 [−0.557, 0.112)	1.43 (0.98, 2.08)	0.062	1.38 (0.95, 2.01)	0.092
61/930	Q3 [−5.520, −0.557)	1.45 (0.99, 2.13)	0.058	1.71 (1.16, 2.51)	0.006
*Female*					
34/913	Continuous, per 1 decrease	1.19 (0.77, 1.83)	0.038	1.07 (0.63, 1.81)	0.805
	Multicategory				
9/301	Q1 [0.115, 1.467]	Reference	Reference	Reference	Reference
11/311	Q2 [−0.394, −0.115)	1.39 (0.58, 3.36)	0.464	1.31 (0.54, 3.18)	0.544
14/301	Q3 [−3.732, −0.394)	1.79 (0.77, 4.15)	0.174	1.34 (0.57, 3.12)	0.501

Abbreviations: CI, confidence interval; HR, hazard ratio.

^a^ Age was used as the covariate in adjusted Cox proportional hazards models.

We investigated the relationship between *η*_basic_ values and CVD risk across various age groups. The cutoff values of age were defined as 50 years by RCS methods, representing the turning points where age was significantly associated with higher CVD risk (Figure S4). Males aged ≥ 50 years with lower *η*_basic_ values were more likely to have a higher risk of CVD (Figure 2B), while CVD risks were not significantly stratified by *η*_basic_ tertiles in both age groups of females (Figure S5). Furthermore, we investigated the prediction performance of *η*_basic_ values on CVD development in different CVD risk subgroups of males (Table S9). Individuals with healthier SBP, DBP, FBG, and H1Ac showed significant associations between VA and CVD risk, while their corresponding risk groups did not.

### 3.2 Association Between Vascular Aging and Traditional CVD Risk Factors

We evaluated the association of the *η*_basic_ values with traditional CVD risk factors by age subgroups in the chronic disease‐healthy dataset without CVD diagnosis. BMI, blood pressure, heart beats, FBG, and TG significantly increased with lower *η*_basic_ values in males across all age subgroups. Furthermore, TC was specific to males with age < 50 years, whereas NLR and ESR were applicable to males with age ≥ 50 years (Table S10). Along with blood pressure, females' BMI, HDL‐C, TG, FBG, and HbA1c were related to the *η*_basic_ values. TC, LDL‐C, and ESR were specific to younger females (age < 50 years), and NLR was specific to older females (age ≥ 50 years) (Table S11). Additionally, we estimated the associations between the *η*_basic_ values with other incident chronic diseases in males (Table S12). Lower *η*_basic_ values were associated with a 57% higher risk of hypertension (HR = 1.57, *p‐*value < 0.001) and a 17% higher risk of hyperlipidemia (HR = 1.17, *p‐*value = 0.008).

### 3.3 Expanded Vascular Age Model Applying to CVD Risk Evaluation

**FIGURE 3:**
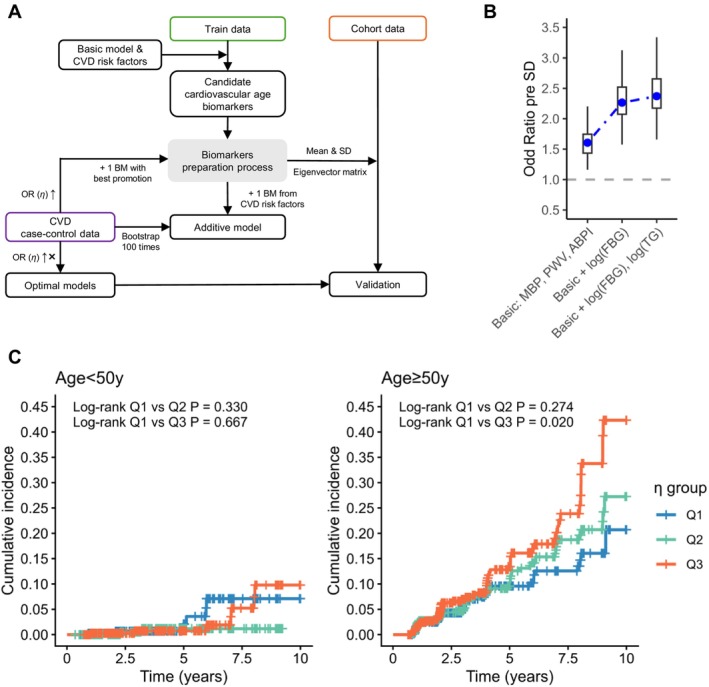
Cardiovascular age expanded model development and performance in males. (A) Scheme for expanded model development and validation. (B) Improvement of *η* across models during iterations. (C) Association between *η*_expand_ and 10‐year CVD incidence in age < 50 years and age ≥ 50 subgroups of males. CVD incidences were observed from three tertiles of *η*_expand_ values: Q1 ranging from [0.029, 2.283], Q2 from [−0.656, 0.029), and Q3 from [−5.102, −0.656). Log‐rank tests were used to compare the differences in CVD incidence between tertile groups.

To improve the performance of the model in CVD risk prediction, we managed to develop an expanded vascular age model in the healthy dataset (Figure 3A). Different traditional CVD risk factors related to the *η* values across age groups were added for the final expanded vascular age models by sex (Figure 3B, Figure S6). FBG and TG were included in the male model, and 4 additional predictors, BMI, HbA1c, FBG, and HDL‐C, were added in females (Tables S13 and S14). Significant correlations between CA and cardiovascular age estimated from the expanded model were also shown in the healthy control dataset (Figure S2B). The association between *η*_expand_ and CVD incidence was evaluated by each gender in the cohort data (Table S15, Figure 3C, Figure S7). Males with lower *η*_expand_ had higher CVD risk after adjusting for age, when FBG and TG improved the model performance in males with age ≥ 50y (C‐index comparison *p‐*value = 0.002). No significant association was observed between *η*_expand_ and CVD development in females, regardless of whether age was included in the model.

## 4 Discussion

In this study, we developed two vascular age models for each sex from people living in Wuhan, China. We calculated the *η* value, a ratio of the Δage value to CA, by the formula (CA‐BA)/CA. The lower value represented a stronger vascular aging trend. The basic model utilizing information on vascular structure and functions showed that people with lower *η* values had higher CVD risk in males. We also evaluated the association between vascular age and traditional CVD risk factors. BMI, blood pressure, FBG, and TG were significantly associated with vascular aging, while other indicators, such as TC, NLR, and ESR, demonstrated age‐specific or sex‐specific relationships. Additionally, we constructed expanded models with additional CVD‐related biomarkers, which mildly improved CVD prediction performance in older males. Here, we want to further examine our models' advantages and their application scenarios.

Age is a well‐known risk factor widely used for CVD prediction, reflecting the contribution of aging to disease development. Besides being a predictor in disease models, some age indexes were also used as indicators to represent cardiovascular health. For example, the FHS developed the heart age index, a different quantification method that estimates the CAs of people with the same risk (D'Agostino Sr. et al. 2008). Previous studies showed that the excess FHS heart ages were associated with a 65% increased risk of cardiovascular disease mortality across populations (Hirsch et al. 2018). Though, this risk age does not necessarily mean biological aging at a functional level (Bafei and Shen 2023).

In contrast, BA is another main type of age index, which reveals the individuals' aging trends and their vulnerability to age‐related disease (Zhao et al. 2024). Aging in CVD could be typically estimated with molecular and cellular biomarkers and functional and structural vascular parameters (Hamczyk et al. 2020). DNAmAge, an epigenetic clock estimated by methylation values of specific CpGs, showed a positive relationship with ischemic stroke and incident CVD (Lind et al. 2018; Soriano‐Tarraga et al. 2016). Other BAs, constructed based on vascular biomarkers, such as arterial age generated by a single coronary artery calcium score (CACS), were also developed to represent functional and structural vascular conditions (McClelland et al. 2009). However, the CACS method leveraged the association between CA and CVD events, and methylation and other molecular‐based methods are impractical in clinical use because of the high cost and limited data accessibility. In this study, we estimated the VA by using the KDM algorithm, which, unlike the traditional multiple linear regression method, using CA as the dependent variable (Klemera and Doubal 2006). This approach avoids relying on biomarkers that are perfectly correlated with CA, which provides little information about the inter‐individual differences in aging. A clinical marker‐derived age acceleration (CAA) was also generated by KDM in a previous study to measure the relationship between accelerated biological aging and disease development (Forrester et al. 2024). This index showed a significant association with disease risk but could not directly reflect vascular conditions. Instead, to explore the effects of vascular aging on CVD risk, we developed basic age models by using baPWV, ABI, and MBP, obtained through non‐invasive approaches, to demonstrate vascular functions and structure. The *η*_basic_ value revealed the vascular aging trend and facilitated comparison across different age groups and other risk groups. Therefore, our *η*_basic_ value, derived from several clinical biomarkers, provided a convenient measure to investigate the roles of vascular aging in CVD development.

Recently, the sex‐specific effects on CVD risk have gained widespread attention, emphasizing the need to consider sex differences in disease mechanisms and prevention strategies (Roeters van Lennep et al. 2023). With this consideration and to dissect the impact of vascular aging on CVD development, we observed variations in the associations of vascular aging with cardiometabolic and inflammatory indicators across different age groups and genders. In males, BMI, TG, and FBG were negatively related to lower *η*_basic_ value, indicating their consistent effects on stronger VA trends in both genders. These results aligned with the previous study, which linked obesity and type 2 diabetes to early VA development in adolescence (Ryder et al. 2020). In older males, NLR and ESR accelerated VA, pinpointing the roles of inflammation contributing to vascular dysfunction, cellular metabolism impairment, and further vascular disease pathogenesis (Ungvari et al. 2018). In females, lipid parameters, TC, HDL, TG, and LDL, were all significantly associated with VA, increasing arterial stiffness (Kilic et al. 2021). Recent findings highlighted lipids in CVD development in women, who experienced worse lipid profiles and were exposed to higher ASCVD risk after menopause transition (Roeters van Lennep et al. 2023). We also observed that *η*_basic_ value tended to decrease with age in females, while males did not show this trend. Previous studies showed that estrogens may have protective effects on arterial stiffness (DuPont et al. 2019). These female sex hormones drop with age, coinciding with the rapid increase in carotid‐femoral PWV (cfPWV) after menopause (Mitchell et al. 2004). Further study also found that postmenopausal females who accepted hormone replacement therapy (HRT) had a lower cfPWV than those who did not (Rajkumar et al. 1997). These results provided insights into sex‐specific relationships between VA and metabolic factors. Because *η* incorporates CA in its calculation, the observed negative association between *η*_basic_ and age in females may partly reflect mathematical coupling rather than true sex‐specific differences in vascular aging. Nonetheless, *η* remains a useful descriptive measure and, together with other analyses, provides insights into potential sex‐specific relationships between vascular aging and metabolic factors. Given the synergistic effects of both risk factors and intrinsic biological aging on CVD, they may shed light on vascular health management strategies and precision disease prevention, such as personalized intervention targeting specific clinical indicators based on individuals' ages and sex (Zhao et al. 2024).

In this study, we utilized multiple datasets from prospective physical examination data to develop vascular age models for each gender. Though we applied these models in both the case–control dataset and cohort dataset where we obtained great performance in CVD prediction, further validation in external data needs to be done in future studies. We estimated the aging acceleration index *η* at baseline and evaluated its relationship with incident CVD. This index does not capture the longitudinal rate of aging. Studies with larger cohorts and repeated follow‐up measurements may help to assess the dynamic impact of aging on disease development. With the limitation of case numbers in females, the vascular age models provided unsatisfactory results in incident CVD prediction. However, the relationships between VA and metabolic factors were worth noting. Larger female datasets, including female‐specific factors such as menopause, hypertensive disorders of pregnancy, and other gynecological indicators and disease histories, need to be collected for model improvement. Finally, this study mainly focused on the Wuhan population. The generalizability of the vascular aging estimates needs to be validated in other independent populations.

In summary, we developed two vascular age models in the general Chinese Wuhan population and explored the subgroup‐specific associations between vascular aging and CVD development and related risk factors. This study provided insight into CVD personalized protection and precision intervention.

## Author Contributions

Tao Li and Cuntai Zhang conceptualized the study. Yueqi Lu, Yucong Zhang, and Bangwei Chen designed the study. Yucong Zhang and Lei Ruan collected the data. Yueqi Lu and Bangwei Chen scrubbed the data. Yueqi Lu developed models and analyzed the data. Yueqi Lu, Yucong Zhang, and Bangwei Chen interpreted the results. Yaxin Li organized the scripts. Yueqi Lu and Li Luo drafted the manuscript. Linpeng Wang, Shida Zhu, and Yutao Du did the critical revision. All authors revised the report and approved the final version before submission.

## Funding

The authors declare that financial support was received for the research and/or publication of this article. This work was supported by the National Key Research and Development Program of China (2020YFC2008002) and Guangdong Province International, Hong Kong, Macao and Taiwan High‐end Talent Exchange Special (2021A1313030024).

## Conflicts of Interest

The authors declare no conflicts of interest.

## Supporting information


**Table S1:** Characteristics of case–control dataset by each gender.
**Table S2:** Characteristics of cohort dataset by each gender.
**Table S3:** Characteristics of healthy training dataset, chronic disease‐healthy dataset, and CVD‐healthy dataset in male after imputation.
**Table S4:** Characteristics of healthy training dataset, chronic disease‐healthy dataset, and CVD‐healthy dataset in female after imputation.
**Table S5:** Characteristics of cohort dataset by each gender after imputation.
**Table S6:** Parameters for Z‐score standardization of biomarkers from cardiovascular age models by sex.
**Table S7:** Eigenvector of biomarkers from cardiovascular age basic model by each gender.
**Table S8:** Parameters of biomarkers used for basic vascular age calculation by each gender.
**Table S9:** Association between *η* from basic model and CVD development by risk factor subgroups in the male cohort dataset.
**Table S10:** Association between *η* from basic model and traditional CVD risk factors by age subgroup in the male chronic disease‐healthy dataset.
**Table S11:** Association between *η* from basic model and traditional CVD risk factors by age subgroup in the female chronic disease‐healthy dataset.
**Table S12:** Association between *η* from the basic model and other incident chronic diseases in males.
**Table S13:** Eigenvector of biomarkers from cardiovascular age expand models by gender.
**Table S14:** Parameters of biomarkers used for expand vascular age calculation by each gender.
**Table S15:** Association between normalized cardiovascular age acceleration index *η*expand and incident CVD by sex.
**Figure S1:** Biomarker‐chronological age trend for standardized CVD risk factors in the healthy dataset by gender.
**Figure S2:** Associations between vascular age and chronological age by sex for the basic model and the expanded model in the healthy control dataset.
**Figure S3:** Associations between *η*basic and chronological age in each sex.
**Figure S4:** Association between chronological age and incident CVD.
**Figure S5:** 10‐year cumulative incidence curve for CVD across different quantile groups of *η*basic in females.
**Figure S6:** Improvement of *η* in nested models from the CVD‐healthy training datasets in females.
**Figure S7:** 10‐year cumulative incidence curves for CVD across different quantile groups of *η*expand in females.

## Data Availability

Data that supports the findings of this paper are available from corresponding authors upon reasonable request. The models used to calculate the normalized cardiovascular age acceleration index are available on the GitHub website (https://github.com/curiousXX/VasAge).
