# Supplementary material for: Estimating Vascular Age to Evaluate the Association Between Aging and Cardiovascular Disease
Source: Aging Cell. 2026 May 5;25(5):e70503. doi: 10.1111/acel.70503 (PMC13143865; doi:10.1111/acel.70503)
Supplement: Supplementary file 1 — Table S1: Characteristics of case–control dataset by each gender. Table S2: Characteristics of cohort dataset by each gender. Table S3: Characteristics of healthy training dataset, chronic disease‐healthy dataset, and CVD‐healthy dataset in male after imputation. Table S4: Characteristics of healthy training dataset, chronic disease‐healthy dataset, and CVD‐healthy dataset in female after imputation. Table S5: Characteristics of cohort dataset by each gender after imputation. Table S6: Parameters for Z‐score standardization of biomarkers from cardiovascular age models by sex. Table S7: Eigenvector of biomarkers from cardiovascular age basic model by each gender. Table S8: Parameters of biomarkers used for basic vascular age calculation by each gender. Table S9: Association between η from basic model and CVD development by risk factor subgroups in the male cohort dataset. Table S10: Association between η from basic model and traditional CVD risk factors by age subgroup in the male chronic disease‐healthy dataset. Table S11: Association between η from basic model and traditional CVD risk factors by age subgroup in the female chronic disease‐healthy dataset. Table S12: Association between η from the basic model and other incident chronic diseases in males. Table S13: Eigenvector of biomarkers from cardiovascular age expand models by gender. Table S14: Parameters of biomarkers used for expand vascular age calculation by each gender. Table S15: Association between normalized cardiovascular age acceleration index ηexpand and incident CVD by sex. Figure S1: Biomarker‐chronological age trend for standardized CVD risk factors in the healthy dataset by gender. Figure S2: Associations between vascular age and chronological age by sex for the basic model and the expanded model in the healthy control dataset. Figure S3: Associations between ηbasic and chronological age in each sex. Figure S4: Association between chronological age and incident CVD. Figure S5: 10‐year cumulative i [file ACEL-25-e70503-s001.pdf]

Estimating vascular age to evaluate the association between aging and cardiovascular disease

Yueqi Lu, Yucong Zhang, Bangwei Chen, Lei Ruan, Yaxin Li, Linpeng Wang, Shida Zhu, TAO LI, Li Luo, Cuntai Zhang, Yutao Du

Supplementary Materials

Contents

Table S1. Characteristics of case-control dataset by each gender. 3

Table S2. Characteristics of cohort dataset by each gender. 4

Table S3. Characteristics of healthy training dataset, chronic disease-healthy dataset, and CVD-healthy dataset in male after imputation. 6

Table S4. Characteristics of healthy training dataset, chronic disease-healthy dataset, and CVD-healthy dataset in female after imputation 8

Table S5. Characteristics of cohort dataset by each gender after imputation 9

Table S6. Parameters for Z-score standardization of biomarkers from cardiovascular age models by sex. 10

Table S7. Eigenvector of biomarkers from cardiovascular age basic model by each gender. 11

Table S8. Parameters of biomarkers used for basic vascular age calculation by each gender. 12

Table S9. Association between η from basic model and CVD development by risk factor subgroups in the male cohort dataset. 13

Table S10. Association between η from basic model and traditional CVD risk factors by age subgroup in the male chronic disease-healthy dataset. 14

Table S11. Association between η from basic model and traditional CVD risk factors by age subgroup in the female chronic disease-healthy dataset. 15

Table S12. Association between η from the basic model and other incident chronic diseases in males. 16

Table S13. Eigenvector of biomarkers from cardiovascular age expand models by gender. 17

Table S14. Parameters of biomarkers used for expand vascular age calculation by each gender. 18

Table S15. Association between normalized cardiovascular age acceleration index η_{expand} and incident CVD by sex 19

Figure S1. Biomarker-chronological age trend for standardized CVD risk factors in the healthy dataset by gender. 20

Figure S2. Associations between vascular age and chronological age by sex for the basic model and the expanded model in the healthy control dataset. 21

Figure S3. Associations between η_{basic} and chronological age in each sex. 22

Figure S4. Association between chronological age and incident CVD 23

Figure S5. 10-year cumulative incidence curve for CVD across different quantile groups of η_{basic} in females 24

Figure S6. Improvement of η in nested models from the CVD-healthy training datasets in females 25

Figure S7. 10-year cumulative incidence curves for CVD across different quantile groups of η_{expand} in females 26

Table S1. Characteristics of case-control dataset by each gender.

Variables	Male (N = 3,177)		Female (N = 1,669)	
	Missing value, n	Values	Missing value, n	Values
Age, years	0	48.41±10.47	0	46.27±11.52
Body mass index, kg/m^2	95	25.38±3.12	28	22.75±3.04
Systolic blood pressure, mmHg	104	126.64±15.94	29	120.70±18.18
Diastolic blood pressure, mmHg	106	83.37±11.34	29	76.16±11.37
*Mean arterial pressure, mmHg	106	97.79±12.18	29	91.00±12.93
Heat beat, beats/min	105	74.14±10.55	31	76.23±10.78
Brachial-ankle pulse wave velocity, cm/s	1,075	1,379.99±251.08	420	1,272.42±280.86
Ankle brachial index	1,076	1.11±0.09	420	1.08±0.08
Cardiovascular disease, n (%)	0	215 (6.77)	0	57 (3.42)
Obesity, n (%)	95	577 (18.72)	28	92 (5.61)
Hyperlipidemia, n (%)	14	1,004 (31.74)	2	249 (14.94)
Hypertension, n (%)	96	1,261 (40.93)	26	361 (21.97)
Diabetes, n (%)	155	353 (11.68)	62	85 (5.29)
Chronic obstructive pulmonary disease, n (%)	0	4 (0.13)	0	1 (0.06)
Thyroid dysfunction, n (%)	0	30 (0.94)	0	52 (3.12)
Lower extremity blockage, n (%)	1,076	21 (1.00)	420	19 (1.52)
Incompressible vascular calcification, n (%)	1,076	7 (0.33)	420	4 (0.32)
Lipid-lowering drugs, n (%)	0	155 (4.88)	0	40 (2.40)
Antihypertensive drugs, n (%)	0	623 (19.61)	0	167 (10.01)
Antidiabetic agent or insulin, n (%)	0	159 (5.00)	0	44 (2.64)
Antiplatelet drugs, n (%)	0	142 (4.47)	0	25 (1.50)
Anticoagulant drugs, n (%)	0	2 (0.06)	0	0 (0)
Nitrates, n (%)	0	4 (0.13)	0	0 (0)
Antiarrhythmia drug, n (%)	0	1 (0.03)	0	0 (0)
Hyperthyroidism drugs, n (%)	0	4 (0.13)	0	3 (0.18)
Hypothyroidism drugs, n (%)	0	23 (0.72)	0	46 (2.76)
Anti-trioxypurine, n (%)	0	30 (0.94)	0	0 (0)
Total cholesterol, mmol/L	4	4.63±0.90	1	4.66±0.93
High-density lipoprotein, mmol/L	17	1.15±0.25	2	1.40±0.30
Low-density lipoprotein, mmol/L	16	2.87±0.78	2	2.82±0.76
Triglyceride, mmol/L	4	1.44 [1.01, 2.14]	1	0.97 [0.71, 1.39]
Fasting blood glucose, mmol/L	4	5.02 [4.64, 5.50]	1	4.80 [4.49, 5.16]
Hemoglobin A1c, %	169	37.71 [35.52, 40.98]	62	36.61 [34.43, 38.80]
*Neutrophil-lymphocyte ratio	7	1.88 [1.39, 2.53]	4	1.82 [1.28, 2.60]
Erythrocyte sedimentation rate, mm/H	298	2.00 [2.00, 5.00]	105	7.00 [5.00, 10.00]

Continuous variables with normal distribution were presented as mean ± standard deviation, continuous variables without normal distribution were presented as median [interquartile range], and categorical variables were presented as frequency (percentage).

*Mean arterial pressure = Diastolic blood pressure + [Systolic blood pressure - Diastolic blood pressure]/3; Neutrophil-lymphocyte ratio = Neutrophils/lymphocyte.

Table S2. Characteristics of cohort dataset by each gender.

Variables	Male (N = 2,819)		Female (N = 913)	
	Missing value, n	Values	Missing value, n	Values
*Cardiovascular disease, n (%)	/	174 (6.17)	/	34 (3.72)
Median follow-up, days	/	1,091	/	762
Age, years	0	50.89±9.76	0	46.96±10.89
Body mass index, kg/m^2	92	25.22±2.78	5	22.68±2.79
Systolic blood pressure, mmHg	141	126.92±14.95	16	119.51±16.21
Diastolic blood pressure, mmHg	141	83.88±10.96	15	76.34±10.74
†Mean arterial pressure, mmHg	141	98.23±11.55	16	90.73±11.83
Heat beat, beats/min	141	74.37±10.07	17	76.49±10.78
Brachial-ankle pulse wave velocity, cm/s	1,533	1,374.37±235.23	369	1,249.19±245.17
Ankle brachial index	1,533	1.11±0.08	369	1.09±0.08
Obesity, n (%)	92	403 (14.78)	5	47 (5.18)
Hyperlipidemia, n (%)	2	818 (29.04)	0	134 (14.68)
Hypertension, n (%)	128	1218 (45.26)	15	201 (22.38)
Diabetes, n (%)	67	319 (11.59)	24	33 (3.71)
Chronic obstructive pulmonary disease, n (%)	0	1 (0.04)	0	0 (0)
Thyroid dysfunction, n (%)	0	17 (0.60)	0	32 (3.50)
Lower extremity blockage, n (%)	1,533	12 (0.93)	369	7 (1.29)
Incompressible vascular calcification, n (%)	1,533	1 (0.08)	369	0 (0)
Lipid-lowering drugs, n (%)	0	91 (3.23)	0	24 (2.63)
Antihypertensive drugs, n (%)	0	545 (19.33)	0	98 (10.73)
Antidiabetic agent or insulin, n (%)	0	138 (4.90)	0	18 (1.97)
Antiplatelet drugs, n (%)	0	68 (2.41)	0	12 (1.31)
Anticoagulant drugs, n (%)	0	1 (0.04)	0	0 (0)
Nitrates, n (%)	0	0 (0)	0	1 (0.11)
Antiarrhythmia drug, n (%)	0	2 (0.07)	0	1 (0.11)
Hyperthyroidism drugs, n (%)	0	4 (0.14)	0	6 (0.66)
Hypothyroidism drugs, n (%)	0	10 (0.35)	0	20 (2.19)
Anti-trioxypurine, n (%)	0	20 (0.71)	0	2 (0.22)
Total cholesterol, mmol/L	0	4.76±0.87	0	4.75±0.87
High-density lipoprotein, mmol/L	2	1.18±0.26	1	1.44±0.30
Low-density lipoprotein, mmol/L	2	2.91±0.74	0	2.84±0.77
Triglyceride, mmol/L	0	1.41 [1.01, 2.08]	0	1.00 [0.71, 1.38]
Fasting blood glucose, mmol/L	0	5.07 [4.72, 5.54]	0	4.82 [4.54, 5.16]
Hemoglobin A1c, %	80	37.71 [35.52, 40.98]	24	36.61 [34.43, 39.89]
†Neutrophil-lymphocyte ratio	1	1.78 [1.31, 2.43]	0	1.81 [1.29, 2.51]
Erythrocyte sedimentation rate, mm/H	201	2.00 [2.00, 4.00]	58	6.00 [4.00, 10.00]

Continuous variables with normal distribution were presented as mean ± standard deviation, continuous variables without normal distribution were presented as median [interquartile range], and categorical variables were presented as frequency (percentage).

*Cardiovascular disease was defined as the first diagnosis during the follow-up physical examination after enrollment.

[†]Mean arterial pressure = Diastolic blood pressure + [Systolic blood pressure - Diastolic blood pressure]/3; Neutrophil-lymphocyte ratio = Neutrophils/lymphocyte.

Table S3. Characteristics of healthy training dataset, chronic disease-healthy dataset, and CVD-healthy dataset in male after imputation.

Variables	Healthy training dataset (N = 828)	Chronic disease-healthy dataset (N = 2,134)	CVD-healthy dataset (N = 550)
Age, years	45.11±10.58	48.60±9.92	49.97±12.30
Body mass index, kg/m^2	23.69±2.33	25.97±3.17	24.33±2.79
Systolic blood pressure, mmHg	116.87±9.76	129.65±16.12	122.59±15.81
Diastolic blood pressure, mmHg	76.47±7.58	85.86±11.51	78.74±9.85
*Mean arterial pressure, mmHg	89.94±7.60	100.45±12.31	93.36±11.11
Heat beat, beats/min	72.33±9.89	74.99±10.73	72.78±10.65
Brachial-ankle pulse wave velocity, cm/s	1,270.85±175.03	1,414.55±250.49	1,334.83±222.23
Ankle brachial index	1.10±0.08	1.12±0.09	1.11±0.09
Cardiovascular disease, n (%)	/	/	215 (39.09)
Obesity, n (%)	/	541 (25.35)	43 (7.82)
Hyperlipidemia, n (%)	/	902 (42.27)	104 (18.91)
Hypertension, n (%)	/	1123 (52.62)	154 (28.00)
Diabetes, n (%)	/	293 (13.73)	63 (11.45)
Chronic obstructive pulmonary disease, n (%)	/	2 (0.09)	2 (0.36)
Thyroid dysfunction, n (%)	/	29 (1.36)	1 (0.18)
Lower extremity blockage, n (%)	/	26 (1.22)	4 (0.73)
Incompressible vascular calcification, n (%)	/	8 (0.37)	1 (0.18)
Lipid-lowering drugs, n (%)	/	86 (4.03)	69 (12.55)
Antihypertensive drugs, n (%)	/	508 (23.81)	115 (20.91)
Antidiabetic agent or insulin, n (%)	/	125 (5.86)	34 (6.18)
Antiplatelet drugs, n (%)	/	59 (2.76)	83 (15.09)
Anticoagulant drugs, n (%)	/	1 (0.05)	1 (0.18)
Nitrates, n (%)	/	1 (0.05)	3 (0.55)
Antiarrhythmia drug, n (%)	/	0 (0)	1 (0.18)
Hyperthyroidism drugs, n (%)	/	4 (0.19)	0 (0)
Hypothyroidism drugs, n (%)	/	22 (1.03)	1 (0.18)
Anti-trioxypurine, n (%)	/	30 (1.41)	0 (0)
Total cholesterol, mmol/L	4.50±0.69	4.74±0.94	4.19±0.78
High-density lipoprotein, mmol/L	1.22±0.25	1.12±0.25	1.20±0.26
Low-density lipoprotein, mmol/L	2.85±0.61	2.93±0.82	2.64±0.70
Triglyceride, mmol/L	1.15 [0.86, 1.55]	1.64 [1.12, 2.46]	1.16 [0.85, 1.57]
Fasting blood glucose, mmol/L	4.84 [4.53, 5.19]	5.07 [4.68, 5.63]	4.98 [4.59, 5.43]
Hemoglobin A1c, %	36.61 [34.43, 38.80]	37.71 [35.52, 40.98]	36.61 [34.43, 39.89]
*Neutrophil-lymphocyte ratio	1.81 [1.35, 2.46]	1.89 [1.40, 2.54]	1.93 [1.43, 2.65]
Erythrocyte sedimentation rate, mm/H	2.00 [2.00, 4.00]	2.00 [2.00, 5.00]	2.00 [2.00, 4.00]

Continuous variables with normal distribution were presented as mean ± standard deviation, continuous variables without normal distribution were presented as median [interquartile range], and categorical variables were presented as frequency (percentage).

[*]Mean arterial pressure = Diastolic blood pressure + [Systolic blood pressure - Diastolic blood pressure]/3; Neutrophil-lymphocyte ratio = Neutrophils/lymphocyte.

Table S4. Characteristics of healthy training dataset, chronic disease-healthy dataset, and CVD-healthy dataset in female after imputation

**Variables**	**Healthy training dataset (N = 779)**	**Chronic disease-healthy dataset (N = 833)**	**CVD-healthy dataset (N = 333)**
Age, years	42.73±10.25	48.63±11.53	43.88±12.68
Body mass index, kg/m^2	21.99±2.49	23.34±3.30	22.02±2.82
Systolic blood pressure, mmHg	113.23±11.62	126.71±20.06	115.53±16.02
Diastolic blood pressure, mmHg	72.05±8.37	79.73±12.45	72.41±9.40
*Mean arterial pressure, mmHg	85.78±8.79	95.39±14.18	86.78±10.87
Heat beat, beats/min	75.38±9.98	77.32±11.38	77.75±12.51
Brachial-ankle pulse wave velocity, cm/s	1,165.95±177.26	1,352.10±311.41	1,203.50±261.89
Ankle brachial index	1.07±0.07	1.09±0.09	1.07±0.08
Cardiovascular disease, n (%)	/	/	57 (17.12)
Obesity, n (%)	/	85 (10.20)	10 (3.00)
Hyperlipidemia, n (%)	/	231 (27.73)	20 (6.01)
Hypertension, n (%)	/	331 (39.74)	33 (9.91)
Diabetes, n (%)	/	76 (9.12)	9 (2.70)
Chronic obstructive pulmonary disease, n (%)	/	1 (0.12)	0 (0)
Thyroid dysfunction, n (%)	/	50 (6.00)	2 (0.60)
Lower extremity blockage, n (%)	/	27 (3.24)	0 (0)
Incompressible vascular calcification, n (%)	/	4 (0.48)	0 (0)
Lipid-lowering drugs, n (%)	/	30 (3.60)	10 (3.00)
Antihypertensive drugs, n (%)	/	142 (17.05)	25 (7.51)
Antidiabetic agent or insulin, n (%)	/	38 (4.56)	6 (1.80)
Antiplatelet drugs, n (%)	/	15 (1.80)	10 (3.00)
Hyperthyroidism drugs, n (%)	/	3 (0.36)	0 (0)
Hypothyroidism drugs, n (%)	/	44 (5.28)	2 (0.60)
Total cholesterol, mmol/L	4.48±0.70	4.83±1.08	4.35±0.73
High-density lipoprotein, mmol/L	1.44±0.28	1.37±0.32	1.45±0.29
Low-density lipoprotein, mmol/L	2.67±0.60	2.98±0.87	2.64±0.64
Triglyceride, mmol/L	0.86 [0.64, 1.14]	1.14 [0.81, 1.66]	0.87 [0.67, 1.19]
Fasting blood glucose, mmol/L	4.73 [4.44, 5.05]	4.86 [4.53, 5.28]	4.69 [4.42, 5.04]
Hemoglobin A1c, %	36.61 [34.43, 38.80]	37.71 [34.43, 39.89]	35.52 [33.33, 37.71]
*Neutrophil-lymphocyte ratio	1.83 [1.28, 2.61]	1.81 [1.29, 2.58]	1.84 [1.37, 2.61]
Erythrocyte sedimentation rate, mm/H	6.00 [4.00, 10.00]	7.00 [5.00, 11.00]	6.00 [4.00, 10.00]

Continuous variables with normal distribution were presented as mean ± standard deviation, continuous variables without normal distribution were presented as median [interquartile range], and categorical variables were presented as frequency (percentage).

*Mean arterial pressure = Diastolic blood pressure + [Systolic blood pressure - Diastolic blood pressure]/3; Neutrophil-lymphocyte ratio = Neutrophils/lymphocyte.

Table S5. Characteristics of cohort dataset by each gender after imputation

Variables	Male (N = 2,819)	Female (N = 913)
[*]Cardiovascular disease, n (%)	174 (6.17)	34 (3.72)
Median follow-up, days	1,091	762
Age, years	50.89±9.76	46.96±10.89
Body mass index, kg/m^2	25.20±2.78	22.67±2.79
Systolic blood pressure, mmHg	126.67±14.82	119.48±16.14
Diastolic blood pressure, mmHg	83.75±10.89	76.31±10.71
[†]Mean arterial pressure, mmHg	98.05±11.45	90.70±11.79
Heat beat, beats/min	74.39±10.13	76.50±10.74
Brachial-ankle pulse wave velocity, cm/s	1,380.56±242.33	1,262.81±258.62
Ankle brachial index	1.11±0.08	1.08±0.08
Obesity, n (%)	416 (14.76)	47 (5.15)
Hyperlipidemia, n (%)	818 (29.02)	134 (14.68)
Hypertension, n (%)	1,243 (44.09)	203 (22.23)
Diabetes, n (%)	320 (11.35)	34 (3.72)
Chronic obstructive pulmonary disease, n (%)	1 (0.04)	0 (0)
Thyroid dysfunction, n (%)	17 (0.60)	32 (3.50)
Lower extremity blockage, n (%)	26 (0.92)	12 (1.31)
Incompressible vascular calcification, n (%)	2 (0.07)	0 (0)
Lipid-lowering drugs, n (%)	91 (3.23)	24 (2.63)
Antihypertensive drugs, n (%)	545 (19.33)	98 (10.73)
Antidiabetic agent or insulin, n (%)	138 (4.90)	18 (1.97)
Antiplatelet drugs, n (%)	68 (2.41)	12 (1.31)
Anticoagulant drugs, n (%)	1 (0.04)	0 (0)
Nitrates, n (%)	0 (0)	1 (0.11)
Antiarrhythmia drug, n (%)	2 (0.07)	1 (0.11)
Hyperthyroidism drugs, n (%)	4 (0.14)	6 (0.66)
Hypothyroidism drugs, n (%)	10 (0.35)	20 (2.19)
Anti-trioxypurine, n (%)	20 (0.71)	2 (0.22)
Total cholesterol, mmol/L	4.76±0.87	4.75±0.87
High-density lipoprotein, mmol/L	1.18±0.26	1.44±0.30
Low-density lipoprotein, mmol/L	2.91±0.74	2.84±0.77
Triglyceride, mmol/L	1.41 [1.01, 2.08]	1.00 [0.71, 1.38]
Fasting blood glucose, mmol/L	5.07 [4.72, 5.54]	4.82 [4.54, 5.16]
Hemoglobin A1c, %	37.71 [35.52, 40.98]	36.61 [34.43, 39.89]
[†]Neutrophil-lymphocyte ratio	1.78 [1.31, 2.43]	1.81 [1.29, 2.51]
Erythrocyte sedimentation rate, mm/H	2.00 [2.00, 4.00]	6.00 [4.00, 10.00]

Continuous variables with normal distribution were presented as mean ± standard deviation, continuous variables without normal distribution were presented as median [interquartile range], and categorical variables were presented as frequency (percentage).
[*]Cardiovascular disease was defined as the first diagnosis during the follow-up physical examination after enrollment.
[†]Mean arterial pressure = Diastolic blood pressure + [Systolic blood pressure - Diastolic blood pressure]/3; Neutrophil-lymphocyte ratio = Neutrophils/lymphocyte.

Table S6. Parameters for Z-score standardization of biomarkers from cardiovascular age models by sex.

Biomarkers	Mean value	Standard deviation
Male		
Mean arterial pressure, mmHg	89.9368	7.5980
Brachial-ankle pulse wave velocity, cm/s	1270.8508	175.0277
Ankle brachial pressure index	1.0991	0.0792
*Fasting blood glucose, mmol/L	1.5758	0.1049
*Triglyceride, mmol/L	0.1247	0.3942
Female		
Mean arterial pressure, mmHg	85.7754	8.7855
Brachial-ankle pulse wave velocity, cm/s	1165.9519	177.2610
Ankle brachial pressure index	1.0722	0.0727
*Hemoglobin A1c, mg/dL	3.5926	0.0845
Body mass index, kg/m^2	21.9855	2.4938
*Fasting blood glucose, mmol/L	1.5540	0.0989
High-density cholesterol, mmol/L	1.4359	0.2812

*Fasting blood glucose, triglyceride, and hemoglobin A1c were transformed by the natural logarithm before standardization.

Table S7. Eigenvector of biomarkers from cardiovascular age basic model by each gender.

Standardized biomarkers	PC1	PC2	PC3
Male			
Brachial-ankle pulse wave velocity	0.686954205	0.115311188	0.717493728
Ankle brachial pressure index	0.307233055	-0.940836333	-0.142950496
Mean arterial pressure	0.658560377	0.318638235	-0.681738884
Female			
Brachial-ankle pulse wave velocity	0.683380036	0.089011659	0.724616209
Ankle brachial pressure index	0.379364645	-0.891311279	-0.248287475
Mean arterial pressure	0.623758120	0.444568475	-0.642872210

Table S8. Parameters of biomarkers used for basic vascular age calculation by each gender.

Variables	q	k	s
Male			
PC1	-1.480848703	0.032827574	1.119412512
PC2	0.247688794	-0.005490785	0.982168680
PC3	-0.538043263	0.011927387	0.801406252
Female			
PC1	-2.099414170	0.049136030	1.090249065
PC2	0.183165996	-0.004286934	0.973860473
PC3	-0.410229231	0.009601267	0.773054902

Table S9. Association between η from basic model and CVD development by risk factor subgroups in the male cohort dataset.

**Subgroups**		**HR (95%CI) per 1 decrease**	**P value***
Systolic blood pressure, mmHg	≥140	0.99 (0.71,1.40)	0.966
	<140	1.39 (1.08,1.79)	**0.010**
Diastolic blood pressure, mmHg	≥90	1.04 (0.75,1.45)	0.793
	<90	1.32 (1.03,1.71)	**0.031**
Fasting blood glucose, mmol/L	≥7.0	0.57 (0.27,1.23)	0.151
	<7.0	1.27 (1.05, 1.53)	**0.014**
Hemoglobin A1c, mg/dL	≥48	0.51 (0.25,1.06)	0.073
	<48	1.27 (1.05,1.54)	**0.015**
Low-density cholesterol, mmol/L	≥4.1	1.58 (0.85,2.91)	0.147
	<4.1	1.18 (0.97,1.43)	0.091
Total cholesterol, mmol/L	≥6.2	1.21 (0.69,2.11)	0.508
	<6.2	1.21 (1.00,1.47)	0.054
Triglyceride, mmol/L	≥2.3	1.10 (0.70,1.71)	0.681
	<2.3	1.22 (0.10, 1.49)	0.053

*Age was used as the covariate in adjusted Cox proportional hazards models.

Table S10. Association between η from basic model and traditional CVD risk factors by age subgroup in the male chronic disease-healthy dataset.

Characteristic	Age subgroup	η_{basic}			P value	P_{trend}
		Q1	Q2	Q3		
N		704	726	704		
Body mass index, kg/m^2	Age<50	25.80 (3.65)	25.82 (3.08)	26.52 (3.18)	**0.003**	**0.003**
	Age≥50	25.55 (2.72)	26.11 (3.13)	25.97 (3.08)	**0.034**	**0.025**
Systolic blood pressure, mmHg	Age<50	118.69 (11.89)	124.79 (12.96)	136.41 (14.42)	**<0.001**	**<0.001**
	Age≥50	123.06 (12.98)	132.37 (13.41)	142.95 (17.21)	**<0.001**	**<0.001**
Diastolic blood pressure, mmHg	Age<50	78.31 (9.31)	84.13 (9.92)	92.51 (11.90)	**<0.001**	**<0.001**
	Age≥50	80.53 (9.12)	87.25 (9.51)	92.41 (10.87)	**<0.001**	**<0.001**
Heartbeat, beats/min	Age<50	74.82 (9.46)	75.36 (10.58)	78.09 (11.11)	**<0.001**	**<0.001**
	Age≥50	72.33 (10.60)	73.39 (10.48)	75.84 (11.19)	**<0.001**	**<0.001**
Total cholesterol, mmol/L	Age<50	4.69 (0.94)	4.83 (0.91)	4.87 (1.01)	**0.026**	**0.022**
	Age≥50	4.65 (0.88)	4.66 (0.88)	4.73 (0.98)	0.513	0.472
High-density cholesterol, mmol/L	Age<50	1.14 (0.25)	1.11 (0.25)	1.09 (0.23)	0.068	**0.014**
	Age≥50	1.12 (0.26)	1.12 (0.24)	1.15 (0.26)	0.206	0.121
Low-density cholesterol, mmol/L	Age<50	2.94 (0.80)	2.97 (0.82)	2.93 (0.88)	0.816	0.693
	Age≥50	2.93 (0.81)	2.88 (0.79)	2.90 (0.84)	0.692	0.608
Triglyceride, mmol/L	Age<50	1.52 [1.01, 2.41]	1.81 [1.16, 2.64]	1.90 [1.24, 2.81]	**<0.001**	**<0.001**
	Age≥50	1.49 [1.01, 2.23]	1.61 [1.12, 2.39]	1.58 [1.16, 2.36]	**0.040**	**0.03**
Fasting blood glucose, mmol/L	Age<50	4.95 [4.54, 5.34]	4.95 [4.58, 5.41]	5.08 [4.72, 5.62]	**<0.001**	**<0.001**
	Age≥50	5.18 [4.77, 5.66]	5.11 [4.75, 5.78]	5.25 [4.80, 6.18]	**0.011**	**0.006**
Hemoglobin A1c, mg/dL	Age<50	36.61 [34.43, 38.80]	36.61 [34.43, 39.89]	36.61 [34.43, 39.89]	0.200	0.075
	Age≥50	38.80 [36.61, 42.08]	38.80 [36.61, 43.17]	38.80 [36.61, 44.26]	0.272	0.158
Neutrophil to Lymphocyte Ratio	Age<50	1.77 [1.35, 2.39]	1.89 [1.45, 2.53]	1.89 [1.41, 2.49]	0.163	0.255
	Age≥50	1.76 [1.24, 2.48]	1.97 [1.43, 2.59]	2.06 [1.57, 2.88]	**<0.001**	**<0.001**
Erythrocyte Sedimentation Rate	Age<50	2.00 [2.00, 4.00]	2.00 [2.00, 4.00]	2.00 [2.00, 4.00]	0.372	0.289
	Age≥50	3.00 [2.00, 5.00]	3.00 [2.00, 5.00]	3.00 [2.00, 6.50]	**0.002**	**0.003**

η_{basic} was divided into three tertiles: Q1 ranging from [-0.101,2.593], Q2 from [-0.776, -0.101), and Q3 from [-9.378, -0.776).

Normally distributed continuous variables were presented as mean (standard deviation), non-normally distributed continuous variables were presented as median [interquartile range].

P values were evaluated by one-way ANOVA test for normally distributed continuous variables or Kruskal-Wallis rank-sum test for non-normally distributed continuous variables. P_{trend} was obtained by Jonckheere-Terpstra trend test for evaluating monotonic changes of variables across η_{basic} tertile groups.

Table S11. Association between η from basic model and traditional CVD risk factors by age subgroup in the female chronic disease-healthy dataset.

**Characteristic**	**Age subgroup**	η_{basic}			**P value**	P_{trend}
		**Q1**	**Q2**	**Q3**		
N		275	283	275		
Body mass index, kg/m^2	Age<50	21.62 (2.96)	22.82 (2.94)	24.31 (3.80)	**<0.001**	**<0.001**
	Age≥50	22.30 (2.75)	24.01 (2.96)	24.94 (3.07)	**<0.001**	**<0.001**
Systolic blood pressure, mmHg	Age<50	108.15 (11.11)	118.37 (11.63)	134.40 (16.95)	**<0.001**	**<0.001**
	Age≥50	115.32 (14.11)	133.00 (14.00)	147.91 (15.37)	**<0.001**	**<0.001**
Diastolic blood pressure, mmHg	Age<50	68.78 (8.42)	77.18 (9.04)	88.70 (10.85)	**<0.001**	**<0.001**
	Age≥50	72.10 (8.97)	82.66 (9.14)	89.41 (10.68)	**<0.001**	**<0.001**
Heartbeat, beats/min	Age<50	80.00 (11.66)	79.31 (11.41)	82.24 (12.70)	0.173	0.283
	Age≥50	73.68 (11.09)	73.51 (9.80)	75.68 (9.87)	0.104	**0.036**
Total cholesterol, mmol/L	Age<50	4.26 (0.89)	4.59 (0.95)	4.73 (0.87)	**<0.001**	**<0.001**
	Age≥50	5.02 (1.12)	5.26 (0.93)	5.16 (1.22)	0.264	0.482
High-density cholesterol, mmol/L	Age<50	1.44 (0.28)	1.38 (0.28)	1.35 (0.33)	**0.031**	**0.002**
	Age≥50	1.45 (0.32)	1.31 (0.33)	1.33 (0.35)	**0.007**	**0.006**
Low-density cholesterol, mmol/L	Age<50	2.61 (0.76)	2.84 (0.81)	2.91 (0.74)	**0.003**	**<0.001**
	Age≥50	3.04 (1.00)	3.25 (0.86)	3.21 (0.85)	0.175	0.166
Triglyceride, mmol/L	Age<50	0.82 [0.63, 1.08]	0.95 [0.73, 1.46]	1.26 [0.88, 1.91]	**<0.001**	**<0.001**
	Age≥50	1.19 [0.90, 1.52]	1.43 [1.09, 2.18]	1.35 [1.00, 1.94]	**0.001**	0.094
Fasting blood glucose, mmol/L	Age<50	4.57 [4.36, 4.85]	4.76 [4.47, 5.07]	4.82 [4.57, 5.22]	**<0.001**	**<0.001**
	Age≥50	4.77 [4.48, 5.22]	5.09 [4.70, 5.62]	5.17 [4.83, 5.83]	**<0.001**	**<0.001**
Hemoglobin A1c, mg/dL	Age<50	34.43 [33.06, 36.61]	35.52 [33.33, 37.71]	36.61 [34.43, 38.80]	**<0.001**	**<0.001**
	Age≥50	37.71 [35.52, 39.89]	39.89 [37.71, 42.08]	40.98 [37.71, 45.36]	**<0.001**	**<0.001**
Neutrophil to Lymphocyte Ratio	Age<50	1.98 [1.40, 2.68]	1.82 [1.28, 2.48]	2.19 [1.64, 2.95]	0.052	0.529
	Age≥50	1.74 [1.27, 2.19]	1.52 [1.13, 2.15]	1.80 [1.30, 2.69]	**0.012**	0.097
Erythrocyte Sedimentation Rate	Age<50	6.00 [4.00, 10.00]	6.00 [4.50, 10.00]	7.00 [5.00, 12.00]	**0.006**	**0.001**
	Age≥50	9.00 [6.00, 11.50]	9.00 [6.00, 12.00]	9.00 [6.00, 12.50]	0.744	0.773

η_{basic} was divided into three tertiles: Q1 ranging from [-0.017,1.879], Q2 from [-0.659, -0.017), and Q3 from [-3.075, -0.659).

Normally distributed continuous variables were presented as mean (standard deviation), non-normally distributed continuous variables were presented as median [interquartile range].

P values were evaluated by one-way ANOVA test for normally distributed continuous variables or Kruskal-Wallis rank-sum test for non-normally distributed continuous variables. P_{trend} was obtained by Jonckheere-Terpstra trend test for evaluating monotonic changes of variables across η_{basic} tertile groups.

Table S12. Association between η from the basic model and other incident chronic diseases in males.

	η_{basic}			
	**Crude**		**Adjusted***	
	**HR†**	**P value**	**HR†**	**P value**
**Hypertension**	1.52	<0.001	1.57	<0.001
**Type II diabetes**	1.19	0.170	1.20	0.162
**Hyperlipidemia**	1.17	0.008	1.17	0.008
**Kidney dysfunction‡**	0.94	0.304	0.97	0.594

*Age was used as the covariate in adjusted Cox proportional hazards models.
†HR was estimated for η value per 1 decrease in each model.
‡Kidney dysfunction was defined as individuals with the estimated glomerular filtration rate (eGFR) < 60 mL/min/1.73m^2.
Abbreviation. HR, hazard ratio; CI, confidence interval.

Table S13. Eigenvector of biomarkers from cardiovascular age expand models by gender.

Standardized biomarkers	PC1	PC2	PC3	PC4	PC5	PC6	PC7
**Male**							
Mean arterial pressure	0.588211242	0.198795173	-0.373132210	-0.011785817	0.689290550	-	-
Brachial-ankle pulse wave velocity	0.594004540	0.322631210	-0.201406358	0.014644965	-0.708723298	-	-
Ankle brachial pressure index	0.249566525	0.398749131	0.870619520	0.011788381	0.143521311	-	-
*Fasting blood glucose	0.348229384	-0.584276287	0.184144836	-0.708350371	-0.041084635	-	-
*Triglyceride	0.342956247	-0.596665679	0.168285959	0.705512158	-0.017421557	-	-
**Female**							
Mean arterial pressure, mmHg	0.426013491	0.154326076	-0.557853926	0.255600482	-0.060929634	-0.255177865	-0.591045849
Brachial-ankle pulse wave velocity, cm/s	0.454743392	0.371268371	-0.249783016	0.092149582	-0.170656188	0.580535410	0.467268923
Ankle brachial pressure index	0.248002424	0.440861878	0.198672775	-0.786930099	-0.158414255	-0.210291982	-0.126838311
*Hemoglobin A1c, mg/dL	0.410152102	0.116793111	0.448322961	0.151649073	0.744258296	0.120612259	-0.160237250
Body mass index, kg/m2	0.458745660	-0.430452183	-0.154030763	-0.107088781	0.127913693	-0.521894298	0.529466037
*Fasting blood glucose, mmol/L	0.313290578	-0.044760232	0.601844737	0.394972945	-0.596528921	-0.150180762	-0.056778569
High-density cholesterol, mmol/L	-0.270910388	0.665620667	-0.000727911	0.341245940	0.126253285	-0.494130490	0.327112419

*Fasting blood glucose, triglyceride, and hemoglobin A1c were transformed by the natural logarithm before standardization.

Table S14. Parameters of biomarkers used for expand vascular age calculation by each gender.

Variables	q	k	s
**Male**			
PC1	-1.591838584	0.035288007	1.158516214
PC2	-0.264038204	0.005853220	1.015758093
PC3	-0.218357044	0.004840557	0.979626129
PC4	0.203564690	-0.004512639	0.927792923
PC5	0.551653949	-0.012229110	0.800404697
**Female**			
PC1	-2.899820294	0.067869247	1.189092801
PC2	-0.573511108	0.013422820	1.072500211
PC3	-0.006808836	0.000159358	1.046390749
PC4	0.099195009	-0.002321623	0.946635475
PC5	-0.637304158	0.014915874	0.836573308
PC6	-0.193210021	0.004522011	0.837143890
PC7	-0.409802710	0.009591284	0.710860734

Table S15. Association between normalized cardiovascular age acceleration index η_{expand} and incident CVD by sex

Case/N		η_{expand}			
		Crude		Adjusted*	
		HR (95% CI)	P value	HR (95% CI)	P value
**Male**					
174/2,819	**Continuous, per 1 decrease**	1.12 (0.94,1.33)	0.189	1.25 (1.04,1.50)	0.015
	**Multicategory**				
47/930	**Q1 [0.029, 2.283]**	Reference	Reference	Reference	Reference
60/959	**Q2 [-0.656, 0.029)**	1.18 (0.80,1.72)	0.405	1.07 (0.73,1.57)	0.719
67/930	**Q3 [-5.102, -0.656)**	1.45 (1.00,2.10)	0.051	1.64 (1.13,2.39)	0.009
**Female**					
34/913	**Continuous, per 1 decrease**	1.65 (0.96,2.86)	0.071	1.77 (0.89,3.54)	0.106
	**Multicategory**				
8/301	**Q1 [0.085,1.384]**	Reference	Reference	Reference	Reference
11/311	**Q2 [-0.278, 0.085)**	1.50 (0.60,3.73)	0.385	1.40 (0.56,3.52)	0.468
15/301	**Q3 [-2.940, -0.278)**	1.79 (0.77,4.15)	0.174	1.64 (0.69,3.89)	0.262

*Age was used as the covariate in adjusted Cox proportional hazards models.
Abbreviation. HR, hazard ratio; CI, confidence interval.

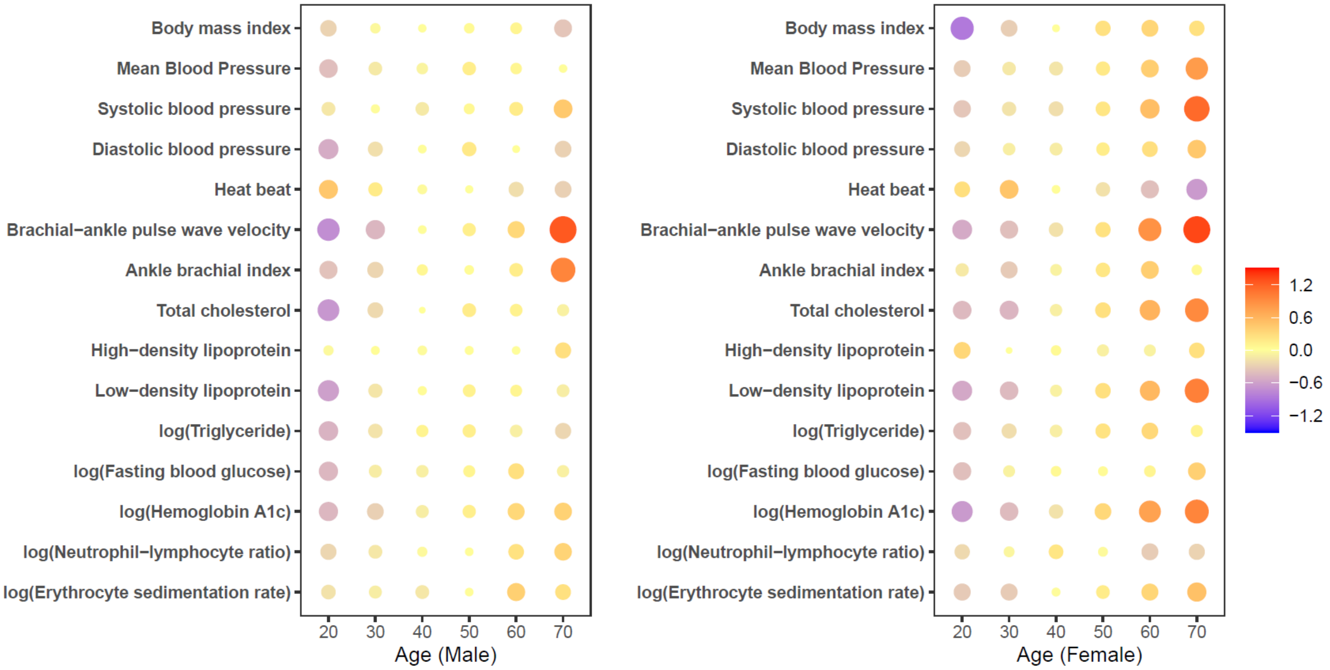

Figure S1. Biomarker-chronological age trend for standardized CVD risk factors in the healthy dataset by gender.

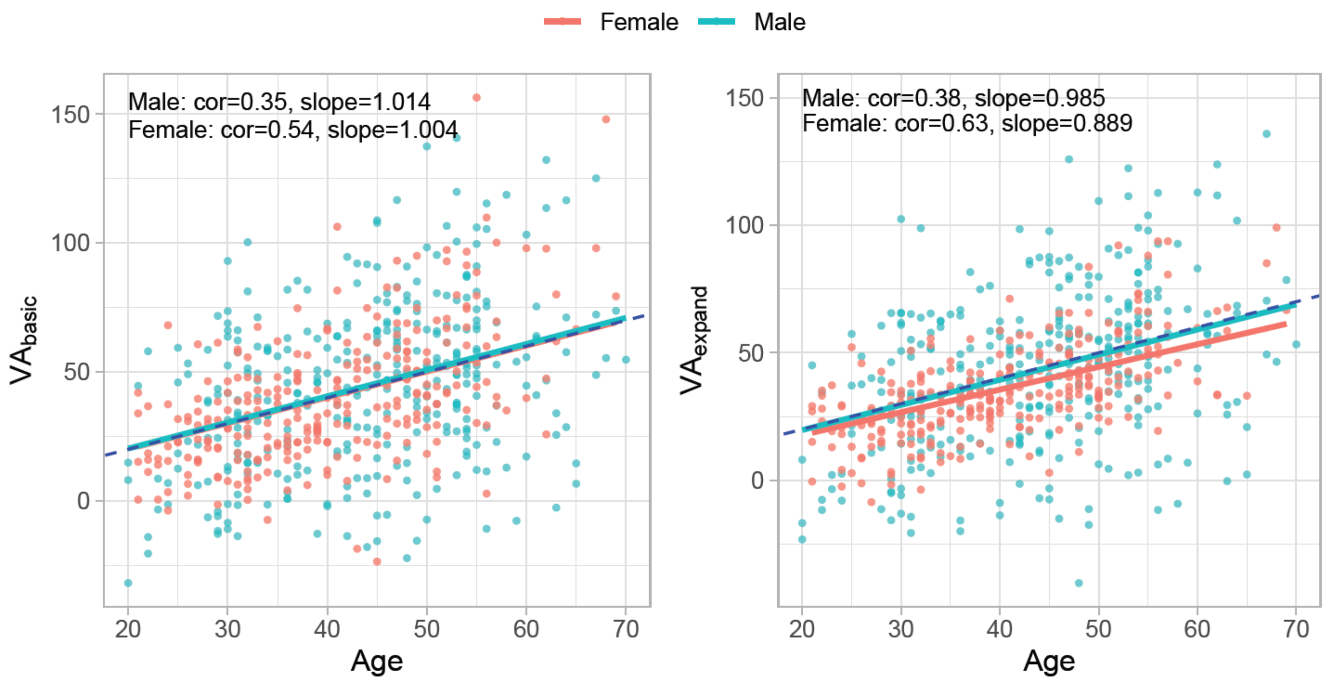

Figure S2. Associations between vascular age and chronological age by sex for the basic model and the expanded model in the healthy control dataset.

The blue dashed line indicates the diagonal where vascular age (VA) equals chronological age (CA). The green and red solid lines show the sex-specific regression of VA on CA (VA = CA + residuals) for males and females, respectively. Cor value denotes the correlation between VA and CA, calculated using the Pearson correlation test. Slope is the regression coefficient of CA on vascular age in a model without an intercept.

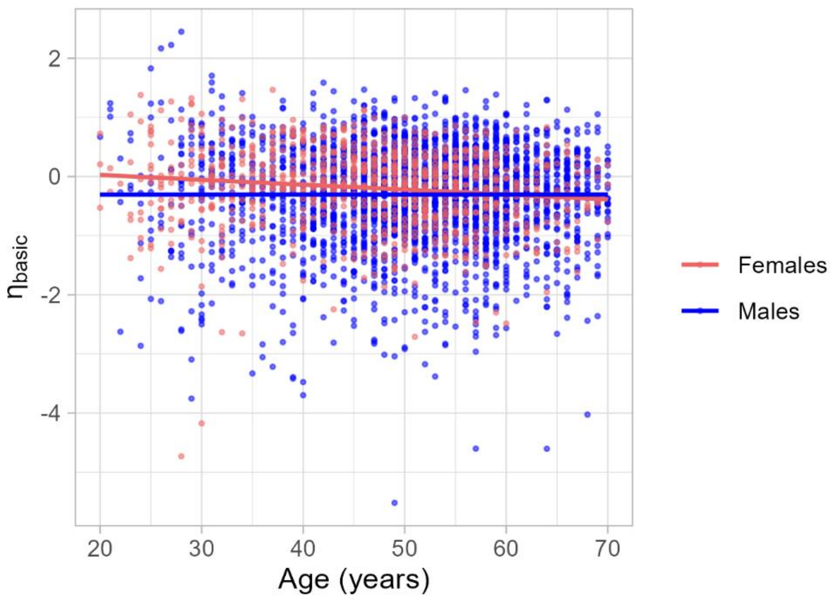

Figure S3. Associations between η_{basic} and chronological age in each sex.

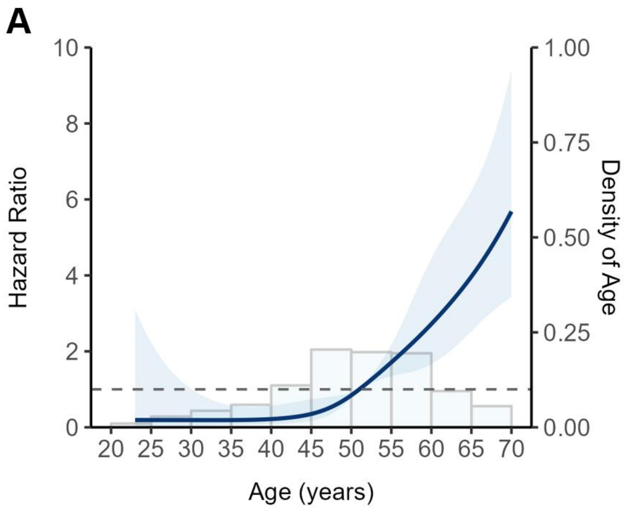

Figure S4. Association between chronological age and incident CVD

Restricted cubic spline (RCS) methods were used to estimate the nonlinear relationship between chronological age and incident CVD in Cox regression models in males (A) and females (B), separately. The RCS were defined with five knots at the 5th, 25th, 50th, 75th, and 95th percentiles.

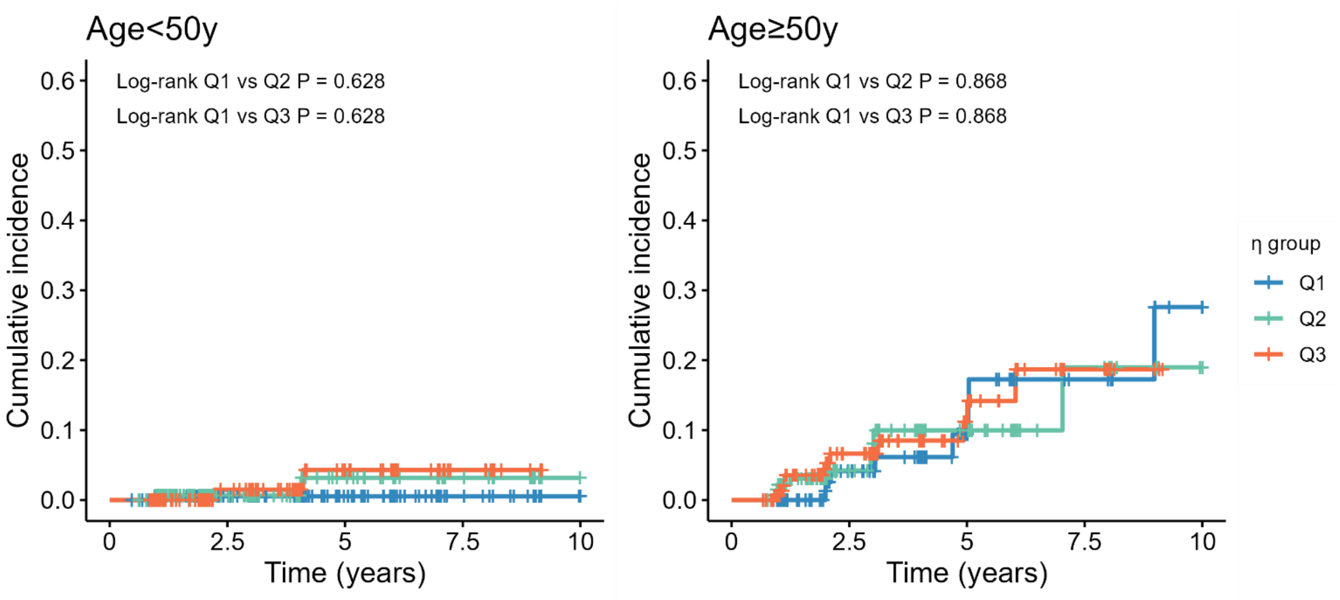

Figure S5. 10-year cumulative incidence curve for CVD across different quantile groups of η_{basic} in females

10-year CVD incidences were observed from η_{basic} three tertiles: Q1 ranging from [0.115,1.467], Q2 from [-0.394, -0.115), and Q3 from [-3.732, -0.394), in females age<50y and age≥50y. Log-rank tests were used to compare the differences in CVD incidence between tertile groups.

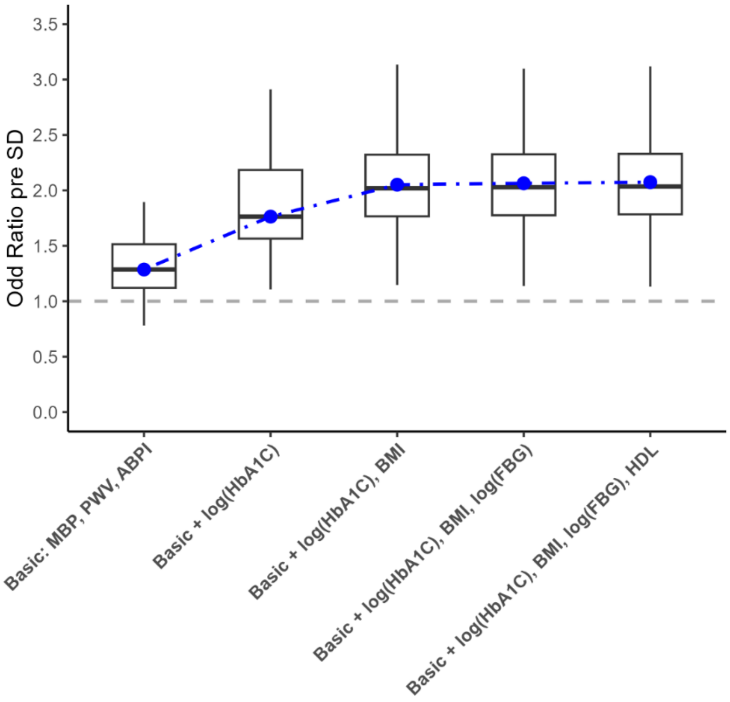

Figure S6. Improvement of η in nested models from the CVD-healthy training datasets in females

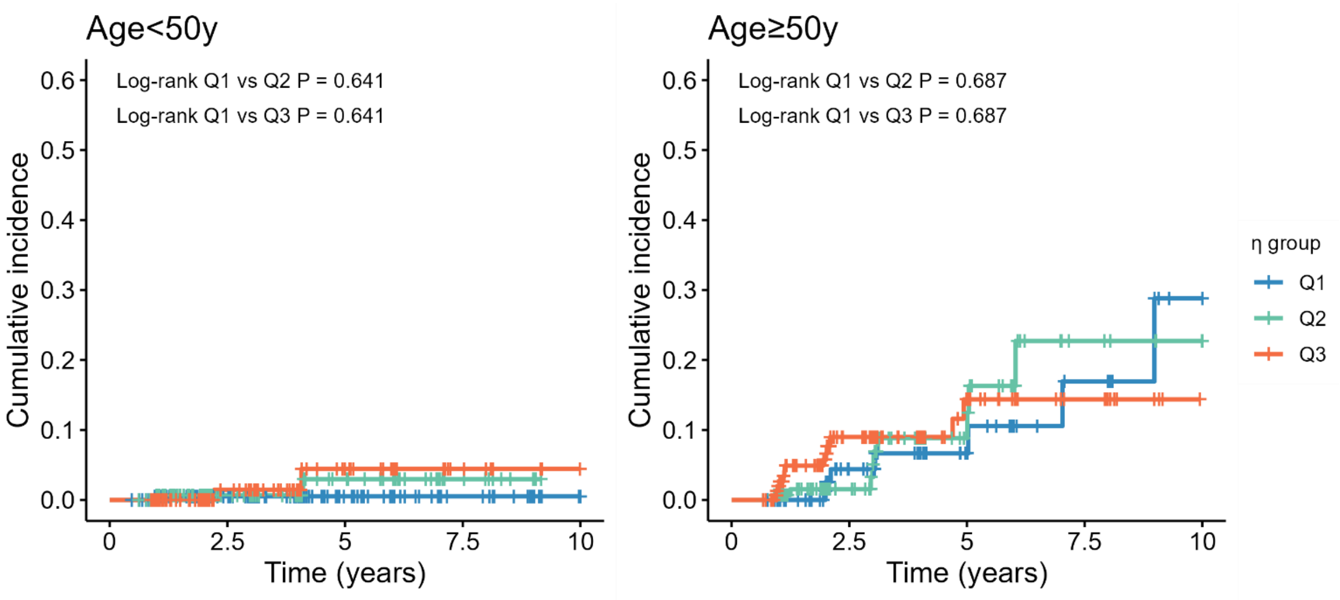

Figure S7. 10-year cumulative incidence curves for CVD across different quantile groups of η_{expand} in females

10-year CVD incidences were observed from η_{expand} three tertiles: Q1 ranging from [0.085,1.384], Q2 from [-0.278, 0.085), and Q3 from [-2.940, -0.278), in females age<50y and age≥50y. Log-rank tests were used to compare the differences in CVD incidence between tertile groups.
